# Factors Associated With Persistent Urinary Incontinence Among Women Undergoing Female Genital Fistula Surgery in the Democratic Republic of Congo From 2017 to 2019

**DOI:** 10.3389/fgwh.2022.896991

**Published:** 2022-06-24

**Authors:** Dolores Nembunzu, Naomie Mayemba, Sidikiba Sidibé, Fassou Mathias Grovogui, Brian Tena Tena Aussak, Don Félicien Banze Kyongolwa, Bienvenu Salim Camara, Vandana Tripathi, Alexandre Delamou

**Affiliations:** ^1^Fistula Clinic, Department of Gynecology and Obstetrics, Saint Joseph Hospital, Kinshasa, Democratic Republic of Congo; ^2^Africa Center of Excellence (CEA-PCMT), University Gamal Abdel Nasser, Conakry, Guinea; ^3^Centre National de Formation et de Recherche en Santé Rurale de Maferinyah, Forécariah, Guinea; ^4^EngenderHealth, Kinshasa, République Démocratique du Congo; ^5^EngenderHealth, New York, NY, United States

**Keywords:** female genital fistula, incontinence, surgical outcome, Democratic Republic of Congo, operational research

## Abstract

**Background:**

Despite high closure rates, residual urinary incontinence remains a common problem after successful closure of a vesico-vaginal fistula. The objective of this study was to identify factors associated with residual urinary incontinence in women with successful fistula closure in sites supported by the Fistula Care *Plus* project in the Democratic Republic of Congo (DRC).

**Material and Methods:**

This was a retrospective cohort study using routine data extracted from the medical records of women undergoing fistula surgery in three hospitals supported by the Fistula Care Plus project in DRC between 2017 and 2019. We analyzed factors associated with residual urinary incontinence among a subsample of women with closed fistula at discharge. We collected data on sociodemographic, clinical, gynecological-obstetrical characteristics, and case management. Univariate and multivariate analyses were performed to determine the factors associated with residual urinary incontinence.

**Results:**

Overall, 31 of 718 women discharged with closed fistula after repair (4.3%; 95% CI: 3.1–6.1) had residual incontinence. The leading causes identified in these women with residual incontinence were urethral voiding (6 women), short urethra (6 women), severe fibrosis (3 women) and micro-bladder (2 women). The prevalence of residual incontinence was higher among women who received repair at the Heal Africa (6.6%) and St Joseph's (3.7%) sites compared with the Panzi site (1.7%). Factors associated with increased odds of persistent urinary incontinence were the Heal Africa repair site (aOR: 54.18; 95% CI: 5.33–550.89), any previous surgeries (aOR: 3.17; 95% CI: 1.10–9.14) and vaginal surgical route (aOR: 6.78; 95% CI: 1.02–45.21).

**Conclusion:**

Prior surgery and repair sites were the main predictors of residual incontinence after fistula closure. Early detection and management of urinary incontinence and further research to understand site contribution to persistent incontinence are needed.

## Introduction

Despite the increase in attended deliveries in developing countries, maternal complications such as female genital fistula are still common (1–3). Social inequalities and inequalities in health care access are significant contributors to maternal morbidity, slowing down developing countries' achievement of international health aims such as Universal Health Coverage and the Sustainable Development Goals (4–6).

Between 16 and 32% of women who have undergone fistula repair surgery in sub-Saharan Africa suffer from residual incontinence (7–11). According to the World Health Organization (WHO), 90% of women with fistula in sub-Saharan Africa live in rural areas where access to health care services is poor (3, 6). In such a context, residual incontinence, which may be perceived as fistula repair failure, represents a heavy burden for the woman and her family, given its social, economic and therapeutic implications (12–15). Several authors report that post-repair residual incontinence results in continued stigmatization and social withdrawal, contributing to ongoing mental distress among women affected by fistula (12, 16–20). The persistent physical disability of incontinence may limit a woman's productivity and, in turn, her quality of life (6, 21).

Factors influencing fistula repair outcomes including residual incontinence are the subject of various investigations. Some researchers and clinicans believe that individual characteristics such as history of fistula repair, partial or total damage to the urethra, size of the fistula, residual bladder size, and presence of severe fibrosis negatively influence fistula repair outcomes (22–25). Other authors argue that structural and organizational factors such as insufficient equipment, inexperience of the surgeon, quality of care and inadequate post-operative monitoring substantially affect fistula repair outcomes (6, 26).

In the Democratic Republic of Congo (DRC), fistula care is free to clients through the efforts of several actors across the country (27, 28). Since 2007, USAID funding through the NGO EngenderHealth has been an important contributor to preventive and surgical care of women with fistula in DRC. Between 2014 and 2020, 3,400 women received fistula care in three care sites through the USAID-funded Fistula Care *Plus* (FC+) project implemented by EngenderHealth. While fistula closure rates and factors associated with successful surgical closure are well-documented (18, 25, 29) less is known about the magnitude and management of residual urinary incontinence after fistula repair. Furthermore, studies documenting fistula management experience to date in DRC relate to non-obstetric fistula or are limited to individual repair hospitals (30, 31).

We sought to more broadly understand the prevalence of residual incontinence after fistula repair and factors associated with its occurrence within the three major fistula repair sites supported by the FC+ project in DRC. This study will provide relevant information to improve the management and quality of care for women with fistula in DRC and similar settings.

## Materials and Methods

### Type and Duration of the Study

This was a retrospective cohort study using routine data abstracted from medical records from three fistula repair hospitals supported by the FC+ Project in DRC between January 1, 2017, and December 31, 2019.

### Study Context

#### General Context

The DRC is a Central African country covering 2.345 million km^2^ with an estimated population of 84.1 million inhabitants in 2018, of which about 70% live in rural areas (32). The country has 515 health zones, and the national health system is decentralized to the provincial level and financed by public and private mechanisms (33). Applying an analysis of 2007 Demographic and Health Survey survey data, it is estimated that the number of women who have experienced fistula is 34,000 (34).

#### Programmatic Context

The USAID-funded Fistula Care *Plus* (FC+) project was implemented in seven countries globally, six of which are in Africa, including DRC.

The study included three of the FC+ supported sites: St. Joseph Hospital in Kinshasa, Panzi General Referral Hospital in Bukavu, and Heal Africa Hospital in Goma. All these sites were implementing the same infection prevention procedures for pre, per and post-surgery (i.e., antibiotherapy) and underwent trainings. However, it is recognized that the experience of surgeons and nursing teams, along with and site-specific contexts might have been different. Those factors were considered as site characteristics and labeled as “site repair.”

***St. Joseph Hospital in Kinshasa*** is the general referral hospital of the Bureau Des Oeuvres Missionnaires (Office of Missionary Works), BDOM) health network. It has a capacity of 300 beds and has existed since 1987 (Table 1).

**Table 1:** Characteristics of care sites supported by the Fistula Care Plus Project in DRC.

**Hospitals**	**Capacity**	**Number of beds dedicated to fistula care department**	**Care team**	**Other characteristics**
Panzi	450 Beds	60 Beds	14 Surgeons (including 4 experts and 1 urologist), 17 nurses, 7 psychologists, 2 physiotherapists, 18 social workers and 2 data managers	A reception center with 32 beds. The site also provides apprenticeships in certain trades to fistula clients.
HEAL Africa	220 Beds	18 Beds	4 Surgeons, 5 nurses, 2 physiotherapists, 2 psychologists and 1 psychosocial counselor	A reception center with 38 beds for women awaiting surgery and rest before returning home. The site integrates the training of fistula clients in different professions.
Saint Joseph	300 Beds	17 Beds	2 Surgeons, 5 nurses, 1 psychologist, 1 physiotherapist	A reception center with 12 beds for women awaiting surgery. The site integrates the training of fistula clients in sewing.

##### Panzi General Hospital

Panzi Hospital is located in the city of Bukavu, South Province/Kivu, DR Congo. From 1999 (date Panzi Hospital was established) to May 22, 2020, 6,957 women with traumatic urogenital and lower digestive fistulas were treated. The site uses three types of fistula classifications: the Waaldjik, Goh, and Panzi scores.

##### HEAL Africa Hospital in Goma

HEAL Africa Hospital is located in the city of Goma in the province of North Kivu and has been in existence since 2000. Fistula treatment is holistic. Treatment is organized in two ways: routinely in the hospital in Goma and through outreach in partner hospitals across the country. The site uses the Waaldjik fistula classification.

### Study Population

**Figure 1:**
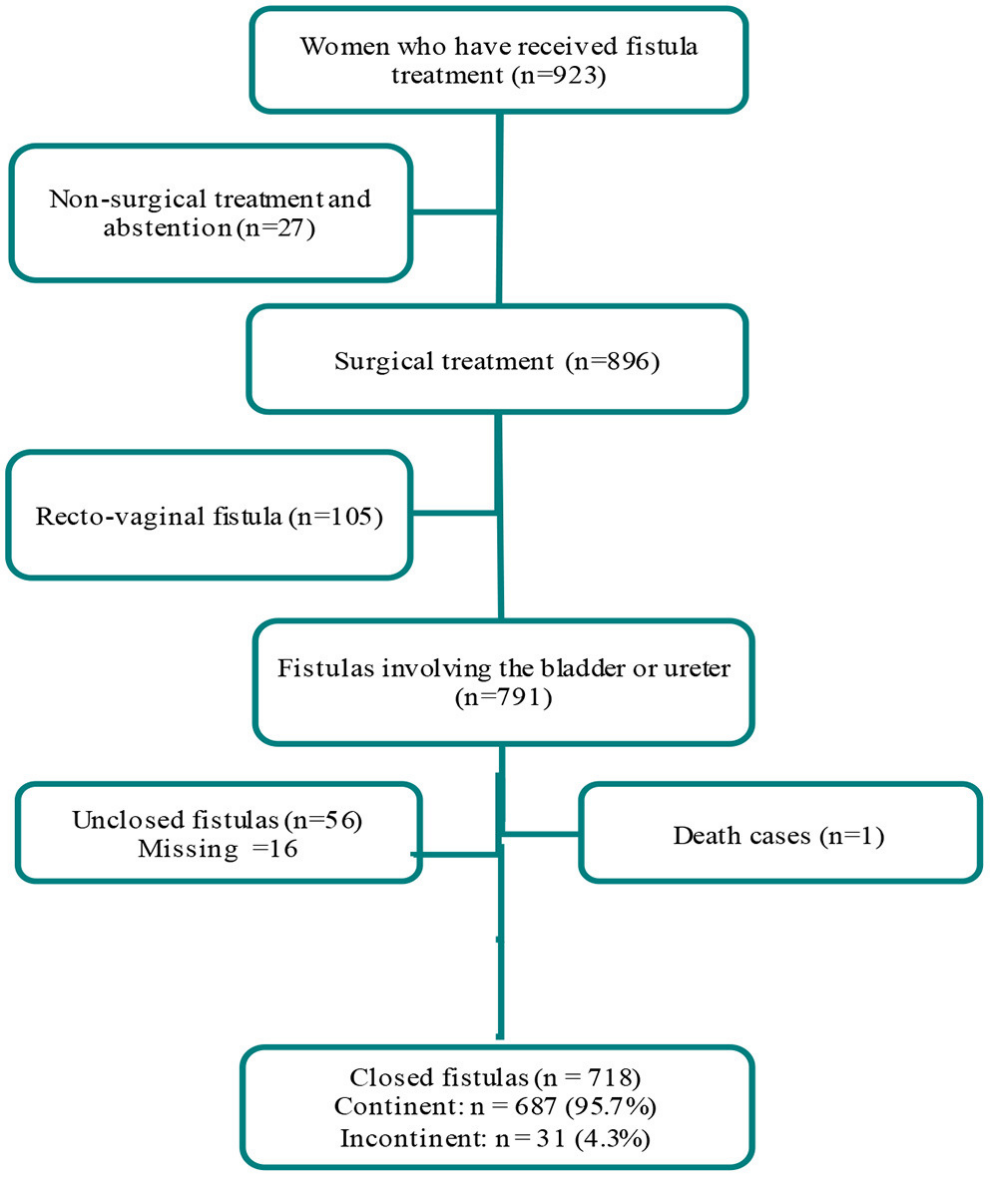
Flow chart of fistula repair patients at sites supported by the Fistula Care *Plus* project in the Democratic Republic of the Congo, 2017–2019.

The study population consisted of women who had undergone surgical repair of urogenital fistula in sites supported by the FC+ project in DRC between January 2017 and December 2019. We limited the analytic sample to only those women who achieved successful fistula closure during repair (*n* = 718; Figure 1).

### Data Collection

Variables related to the study's objectives were extracted from patients' medical records at fistula repair sites, using questionnaires developed using KoBoCollect software.

### Measures

The dependent variable was the occurrence of residual incontinence in women with closed fistula after repair surgery. The independent variables included sociodemographic characteristics (age at admission, marital status, level of education, occupation, and area of residence), clinical characteristics (number of incontinence operations, cause and treatment of incontinence and the operating technique used, among others), parity, and length of stay. Fistula repair was called successful when the woman had a fistula closed and continent at the time of the assessment.

### Data Analysis

The data collected from patient records were analyzed using Stata 16 software (Stata Corporation, College Station, TX, USA). We described patient characteristics through calculating frequencies (%) and means (with standard deviation). Bivariate analyses were performed to explore factors associated with residual incontinence after fistula repair. Student's *t*-test was used to test for differences in continuous variables (age, number of previous surgeries, parity and length of stay or duration of catheterization) and Chi-square test for the remaining, categorical variables. Multivariable logistic regression was used to calculate unadjusted and adjusted odds ratios. The variables which had a *p* ≤ 0.20 in bivariate analyses were included in the logistic regression model. Differences were considered statistically significant where *p* ≤ 0.05.

### Ethical Considerations

The Ethics Committee of the School of Public Health of Kinshasa approved the study protocol (ESP/CE/153/2020).

## Results

### Sociodemographic, Clinical and Gynecological Characteristics

A total of 718 women had successful fistula closure at hospital discharge. Figure 1 shows the selection process and criteria for our sample selection. The sociodemographic, clinical, and gynecological-obstetric characteristics of women included within our analytic sample are presented in Table 2 Patients' mean age was 35 ± 0.5 years. The majority of the women were married or in union (58.7%), housewives or farmers (68.5%), and lived in rural areas (82.6%). More than one-third (35.5%) reported no formal education. The average parity among women was four children per woman (4 ± 3 children).

**Table 2:** Sociodemographic and clinical characteristics of patients who underwent fistula repair in sites supported by the Fistula Care Plus project in the Democratic Republic of Congo, 2017–2019.

**Socio-demographic characteristics**	***n* = 718**	**%**
**Mean age (Standard deviation)**	35 ± 12.6	
**Marital status**		
Married/In union	422	58.8
Not in union	277	38.6
Missing	19	2.7
**Occupation**		
None/student	81	11.3
Housewife	152	21.2
Grower	340	47.4
Civil servant/liberal	73	10.2
Missing	72	10.0
**Level of education**		
None	255	35.5
Primary	247	34.4
Secondary	176	24.5
Missing	40	5.6
**Residence**		
Rural	593	82.6
Urban	122	17.0
Missing	3	0.4
**Site**		
HEAL Africa	209	29.1
Panzi	291	40.5
St joseph	218	30.4
**Clinical characteristics**		
**Delivery mode (*****N*** **=** **632)**		
vaginal birth	254	40.2
Cesarean section	368	58.2
Unknown	10	1.6
**Parity**		
Average number (SD)	4.1 ± 2.9	
**Cause of the fistula (*****n*** **=** **624)**		
Prolonged labor	131	21.0
Obstructed labor	191	30.1
Cesarean section	288	46.2
Others^a^	14	2.2
**Duration of fistula**		
≤1 year	252	35.1
2–4 years old	139	19.4
≥5 years	270	37.6
Unknown	57	7.9
**Types of fistula**		
Uretero-vaginal fistula	46	6.4
Vesico vaginal fistula	556	77.4
Vesico-uterine fistula	88	12.3
Others	6	0.8
**Fistula size in centimeters (cm)**		
Small (<1.5 cm)	234	32.6
Medium (1.5–3 cm)	320	44.6
Large (4 and more)	114	15.9
Unknown	50	7.0
**Number of previous surgeries**		
Mean number (SD)	0.68 ± 0.4	
0	409	57.0
1	205	28.7
2	63	8.8
≥3	40	5.6
Missing	1	0.1
**Waaldjiik classification**		
Type I	374	52.1
Type II	159	22.1
Type III	178	24.8
Classification errors	7	1.0
**Surgical route of repair**		
Vaginal	515	71.7
Abdominal	191	26.6
Abdominal vaginal	10	1.4
Missing	2	0.3
**Duration of fistula repair hospitalization (in days)**		
Mean number (SD)	23.0 ± 0.7	

a *Symphsiotomy, episiotomy, hysterectomy*.

Of the 632 women for whom information was available on delivery mode for the delivery resulting in the fistula, 368 (58.2%) delivered by cesarean section. Most women (46.2%) developed fistula due to cesarean section and prolonged labor (30.1%), and 270 women (37.6%) had a fistula duration of more than 4 years prior to repair. Vesicovaginal fistula was the most common type of fistula (77.4%) and 44.6% of women had a fistula size between 1.5 and 3 cm. Among included women, 308 women (42.9%) had any previous fistula repair surgeries. In terms of case management, the vaginal route was the primary surgical approach for 515 women (71.7%), and average length of stay was 23 days in hospital (Table 2).

### Frequency of Residual Incontinence

Overall, 31 of 718 women (4.3%) had residual incontinence (Table 3). Among these 31 women, information on surgical care was available for 26. 11 of 26 women had incontinence after the first fistula repair and 10 after at least two surgical repairs. The main causes of incontinence identified were urethral incontinence (six cases), short urethra (six cases), severe fibrosis (three cases) and micro-bladder (two cases). Information of incontinence cause was missing for nine women. As treatment, 10 of these 26 women underwent perineal muscle rehabilitation, nine underwent reconstructive surgery, and information was missing for seven (Table 3).

**Table 3:** Profile of care for women with residual incontinence among women who have benefited from fistula closure in sites supported by the Fistula Care Plus project in the Democratic Republic of the Congo from 2017–2019 (*n* = 26).

**Variable**	***N* (26)**	**%**
**Time the incontinence was discovered after repair**		
Unknown	6	23.08
First appointment	13	50
Second and up	3	11.54
Missing	4	15.38
**Circumstances in which incontinence occurred**		
Walk	4	15.38
Stress? (coughing, laughing)	4	15.38
In bed	4	15.38
Any time	8	30.77
Missing	5	19.23
Urgent urination	1	3.85
**Cause of incontinence**		
Urethra voiding	6	23.08
Severe vaginal fibrosis	3	11.54
Micro bladder	2	7.69
Short urethra	6	23.08
Missing	9	34.62
**Incontinence treatment**		
Surgery	9	34.62
Rehabilitation of perineal muscles	10	38.46
Missing	7	26.92

**Figure 2:**
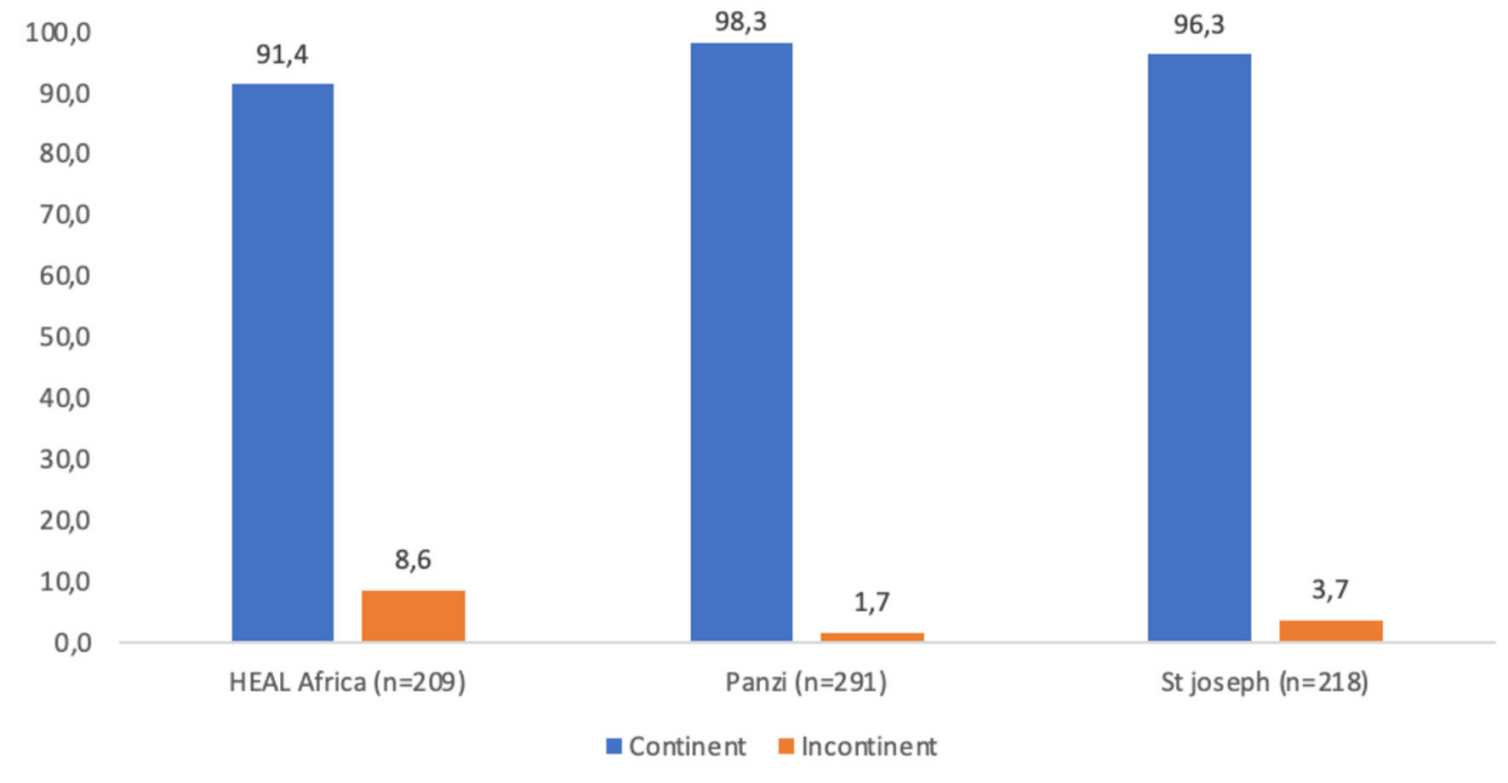
Prevalence of residual urinary incontinence by fistula repair site among sites supported by the Fistula Care Plus project in the Democratic Republic of Congo, 2017–2019.

The frequency of residual incontinence was higher among women who underwent repair at the Heal Africa (18 cases; 8.6%) and St. Joseph's (8 cases; 3.7%) sites compared to the Panzi site (5 cases; 1.7%) (Figure 2).

### Factors Associated With Residual Incontinence in Women With Successful Fistula Closure

In bivariate analyses, parity, site, previous surgical repair, mode of delivery, number of prior surgeries, surgical route and length of hospital stay were significantly associated with residual urine incontinence after fistula repair (Table 4). However, in the multivariate logistic regression (Table 4), after adjusting for other variables, only the fistula repair site, number of previous surgeries, and surgical route remained independently associated with residual incontinence. Compared to women undergoing care at Panzi Hospital, those operated on by Heal Africa faced an over forty-fold increased adjusted odds of residual incontinence (aOR: 43.57; 95% CI: 4.26–445.26).

**Table 4:** Factors associated with residual urinary incontinence in women who had successful fistula closure at sites supported by the Fistula Care Plus project in the Democratic Republic of Congo, 2017–2019.

	**OR**	**95% CI**		***P*-value**	**AOR**	**95% CI**		***P*-value**
**Characteristics**		**Lower**	**Upper**			**Lower**	**Upper**	
Age	0.99	0.96	1.03	0.644	1.00	0.92	1.07	0.913
**Marital status**								
Married/In union	1.00				1.00			
Single, Separated/divorced	1.11	0.53	2.29	0.788	1.20	0.41	3.54	0.745
**Educational attainment**								
None	1.65	0.62	4.36	0.317				
Primary	1.32	0.48	3.64	0.591				
Secondary/Higher	1.00							
**Occupation**								
None/student	1.00							
Housewife	1.83	0.48	6.85	0.369				
Farmer	1.03	0.28	3.71	0.960				
Civil servant/liberal	0.73	0.11	4.51	0.737				
**Residence**								
Rural	1.00				1.00			
Urban	1.74	0.76	3.98	0.191	1.56	0.47	5.20	0.471
**Parity**								
Mean	0.83	0.71	0.97	0.022	0.88	0.68	1.14	0.351
**Site**								
HEAL Africa	5.39	1.97	14.67	0.001	54.18	5.33	550.89	0.001
Panzi	1.00				1.00			
St Joseph	2.18	0.70	6.76	0.177	9.54	0.90	101.15	0.061
**Mode of fistula-causing delivery**								
Vaginal	2.57	1.15	5.71	0.021	2.47	0.83	7.33	0.103
Cesarean section	1.00				1.00			
**Duration of the fistula**	1.01	0.96	1.06	0.732	1.07	0.97	1.18	0.179
**Types of fistula**								
Vesico-vaginal fistula	0.83	0.36	1.89	0.659	0.29	0.07	1.25	0.096
Other fistulas	1.00							
**Fistula size in centimeters (cm)**								
Small (<1.5 cm)	1.00				1.00			
Medium (1.5–3 cm)	0.61	0.27	1.38	0.231	1.14	0.36	3.58	0.829
Large (4 and more)	0.62	0.19	1.94	0.410	2.53	0.57	11.21	0.221
**Number of previous surgeries**								
0	1.00				1.00			
≥1	2.51	1.19	5.33	0.016	3.17	1.10	9.14	0.033
**Surgical route**								
Abdominal/abdomino-vaginal	1.00				1.00			
Vaginal	2.08	0.79	5.51	0.138	6.78	1.02	45.21	0.048

We found that the likelihood of developing residual incontinence was three times higher among women who had any previous fistula repair surgery than those who had not (AOR: 3.17, 95% CI: 1.10–9.14). Compared to women who had an abdominal surgical route of repair, those with a vaginal surgical route of repair were about seven times more likely to experience residual incontinence (AOR: 6.78, 95%CI: 1.02–45.21).

## Discussion

This study provides insight into the frequency of and factors associated with residual incontinence after genital fistula closure among women who have undergone repair in three fistula care sites in DRC. The frequency of residual urinary incontinence was low following surgery. In multivariable analyses, fistula repair site and the number of previous surgeries remained independently associated with residual incontinence.

### Frequency of Residual Incontinence

We found 4.3% of women experienced residual urinary incontinence, which is substantially lower than rates reported elsewhere in sub-Saharan Africa which range from 5 to 71% (18, 25, 35–38). This could be explained by the level of experience of surgical teams at these sites, as well as the FC+ project's support through technical assistance, particularly surgical training and equipment provision.

### Factors Associated With Residual Incontinence in Women With Fistula Repair

We found that the frequency of residual incontinence varied according to the fistula repair sites, with residual incontinence less frequent among women operated on at the Panzi site than at the other two sites, despite adjustment for several factors reflecting fistula severity. A contrary result was reported by Delamou et al. (18), who found that the surgical site was not associated with residual incontinence in women (18). In our context, the differing expertise and the number of surgeons across the different sites might explain such results.

In the literature, the extent of urethral injury, tissue loss of the bladder neck/posterior urethra, overlying pubo-cervical fascia, circumferential fistulae and severe fibrosis have been reported as anatomical causes related to the occurrence of residual incontinence in women after repair (39). In our study, residual incontinence occurred among women with anatomical risk factors including urethral gap, short urethra, and severe fibrosis. We also found that having any previous fistula repair surgery was associated with a three-fold increase in residual incontinence. Similar results were reported by Kimassoum et al. (19), who found that out of 84 patients, 36 women experienced residual incontinence after the second fistula repair and 25 women experienced it after three or more fistula repairs (19). The findings also showed a significant relationship between vaginal surgical route and residual incontinence after repair. Further investigation will be needed to document better this phenomenon and guide practitioners on appropriate intervention among incontinent women after surgical fistula repair.

We found no sociodemographic characteristics to be associated with residual incontinence after repair; this finding is consistent with what has already been reported in the literature (18, 24, 25, 29, 40).

Furthermore, parity and mode of fistula causative delivery were not independently associated with residual urinary incontinence after fistula closure. Other studies have reported an association between parity and residual incontinence in women after repair (35, 41, 42). Finally, we did not find an association between certain fistula characteristics, including fistula size and duration of fistula, with residual incontinence in women after fistula closure. Fistula size was not independently predictive of residual incontinence in women after fistula closure in the published literature (18, 25, 43, 44).

Our study is one of the first to examine the factors associated with residual incontinence after fistula closure in women treated in the three main repair sites supported by FC+ in DRC. All three sites are referral hospitals specialized in fistula treatment with experience in both routine care and outreach. The three sites are assisted by the same external development partner for provision of training, equipment and technical support. The nearly equal distribution of the sample across the three included facilities is also an asset to the study's sample, representing the geographic diversity of repair sites: the St. Joseph site in the west, while Panzi and Heal Africa are located in eastern DRC. Furthermore, outreach repair campaigns are conducted by all the sites, allowing for full national coverage, especially those areas where women are most vulnerable.

The findings of this study should be interpreted with caution given some limitations. Due to the retrospective nature of our study, we are missing data on certain women's characteristics. We were also limited in the selection of variables which could be included within our analysis, and it is possible that not all confounding factors were examined or controlled for. For instance, we could not account for the cause of the fistula even though Cesarean section (CS) was reported as the leading cause of fistula occurrence. Yet, we acknowledge the fact that some women who had a prolonged labor or obstructed labor underwent CS and subsequently had a fistula. The fact that this information was not recorded in the registers might have overestimated CS related causes. This represents a limitation of the study which relied on routine data and calls for the systematic inclusion and collection of such information in medical records.

Additionally, the small number of sites did not allow us to explore the influence of site-level variables; for example, the number of qualified surgeons was not spread evenly across the three repair sites, which may have been an important factor responsible for differences in outcomes across sites. The number of women with residual incontinence is also small, which limited the statistical power for certain comparisons. Finally, the diagnosis of residual incontinence was not possible in patients who were lost to follow-up, i.e., could not show up for their 3-months post-repair visit.

## Conclusion

This study shows that prior surgery and repair sites were independent predictors of residual incontinence after fistula closure with our study sites. These findings highlight the need for medium and long-term follow-up of women who have undergone fistula repair surgery for the detection and proper management of residual incontinence. Besides, there is a need to train additional surgeons for equitable capacity across sites. Developing a better understanding of the contextual factors contributing to residual incontinence after fistula closure through continued research may better inform current and future fistula programs and policies.

## Data Availability Statement

The raw data supporting the conclusions of this article will be made available by the authors, without undue reservation.

## Ethics Statement

The studies involving human participants were reviewed and approved by Ethics Committee of the School of Public Health of Kinshasa. Written informed consent for participation was not required for this study in accordance with the national legislation and the institutional requirements.

## Author Contributions

The study protocol was developed by DN, NM, and BA and reviewed by SS and AD. Data collection was ensured by DN, NM and FG did the data analysis. The first draft of the manuscript was written by par DN and NM and critically reviewed by SS and AD. All authors were involved with interpretation, read, and agreed to the final version of this manuscript.

## Funding

The study and related manuscript development were funded by the United States Agency for International Development (USAID) under associate cooperative agreements AID-OAA-A14-00013 and 7200AA20CA00011.

## Author Disclaimer

The opinions expressed are those of the authors and do not necessarily reflect the views of USAID, or the United States Government.

## Conflict of Interest

BA, DB, and VT were employed by EngenderHealth. The remaining authors declare that the research was conducted in the absence of any commercial or financial relationships that could be construed as a potential conflict of interest.

## Publisher's Note

All claims expressed in this article are solely those of the authors and do not necessarily represent those of their affiliated organizations, or those of the publisher, the editors and the reviewers. Any product that may be evaluated in this article, or claim that may be made by its manufacturer, is not guaranteed or endorsed by the publisher.
